# Development of *in vitro - in vivo* correlations for newly optimized Nimesulide formulations

**DOI:** 10.1371/journal.pone.0203123

**Published:** 2018-08-31

**Authors:** Muhammad Hanif, Muhammad Harris Shoaib, Rabia Ismail Yousuf, Farya Zafar

**Affiliations:** 1 Department of Pharmaceutics, Faculty of Pharmacy, Bahaudin Zakaria University Multan, Punjab, Pakistan; 2 Department of Pharmaceutics, Faculty of Pharmacy and Pharmaceutical Sciences, University of Karachi, Sindh, Karachi, Pakistan; University of South Alabama Mitchell Cancer Institute, UNITED STATES

## Abstract

Use of the human volunteers in bioequivalence studies is being discouraged by the Food and drug administration after the introduction of biowaiver approaches. *In-vitro in-vivo correlation* (*IVIVC*) with the level A is accepted for the registration of new molecules. In the present study deconvolution technique with numeric approaches was applied after compressing and *in vitro* validating the 100mg Nimesulide immediate, intermediate and slow release tablets. Single centered, crossover, randomized study was conducted in four phases with a two-week washout period to obtain the plasma drug concentration data after administrating test and reference products in male healthy volunteers. Kinetica^TM^ 4.4.1 (Thermoelectron corp, USA) was used for the calculation of two ways ANOVA with 90% CI from both log transformed and non- transformed data and Phoenix WinNonlin 7 and it's *IVIVC* toolkit version 7.0 was used for the application of numeric approaches of *IVIVC*. Results revealed that the individual internal percentage prediction error for AUC_*inf*_ and C_*max*_ were found to be < 15% while their average values were < 10% in all medium. Numeric values of % PE at pH 6.8 and pH 7.4 (50 rpm in USP II and 100 rpm in USP I and II apparatus) were found to be (2.5842, 2.9789 and, 7.1732; 7.0944, 2.4721 and 4.350) for AUC_*inf*_ and (2.5842, 0.5736 and 4.6928; 5.6214, 3.0551 and -2.4711) values for C_*max*_ respectively. The low values of prediction errors demonstrate that the correlation model is projecting the *in vivo* response of each formulation. Percentage External error (% PE) was not required because individual values of percentage internal error (%PE) of C_*max*_ and AUC_*last*_ were not >15. In order to predict point to point correlation between fraction drug dissolved and drug absorbed, their mean *r*^*2*^ value was found to be > 0.9112 which showed a linear correlation in slightly alkaline pH.

## 1. Introduction

Bioequivalence studies are considered very much important for the establishment of new generic dosage forms. It is a significant method to estimate the *in vivo* performance of the compound which can be used as a surrogate to determine the therapeutic efficacy [1]. Due to the extensive availability of generic compounds locally in the market, the need for bioequivalence studies are much more required as compared to the past decade [2]. Use of the human being for the bioequivalence studies, cost of the study, subject to subject variation, unavailability of the expertise for the bioequivalence studies made them more complicated especially in case of generic product development. Pharmaceutical scientists have an urge to develop such techniques which are not only cost-effective but also useful for the establishment of generic products.

*In vitro*-*in vivo* correlations (*IVIVC*) plays a significant role in the product development and optimization process which is a very time consuming and costly procedure. Optimization of formulation requires modification in the composition of formulation, batch sizes and equipment and manufacturing procedures. If one or more such alterations are carried out in the formulation, then *in vivo* studies are required to be conducted to prove the comparison of the new formulation with a conventional product which will increase the total optimization cost and also increase the expenditure of carrying out multiple bioequivalence studies. In order to avoid these problems, *IVIVC* studies are carried out for the development of the pharmaceutical product [3, 4]. Several studies demonstrating the utilization of *in vitro* dissolution assessment as success criteria for the prediction of the bioavailability studies [5]. *IVIVC* not only reduces the time and also minimizes the *in vivo* experiments, but also recommended for regulatory purposes. Scientists reported that successful correlation can be used as a surrogate for bioequivalence studies and to support the biowaiver studies. Such studies can be useful to develop suitable dissolution specifications [6]. *IVIVC* is also adequate for the rationalization of therapeutically significant drug release specifications of the formulations [7].

Following FDA guidelines, four levels of *IVIVC* i.e. Level A, B, C and multiple C were present. Correlation level depends upon the ability of the correlation to demonstrate the plasma level profile completely, which may due to the administration of the given dosage form. Pharmacokinetic studies are considered very significant in the development of innovator dosage form but found to be the most expensive task for the pharmaceutical company. Three different types of guidelines have been established by the Food and Drug Administration (FDA) for biowaiver studies of all types of drugs. First one is about the release pattern if the dosage forms show more than 85% release of drug within 30min it will be accepted without *in vivo* studies. The second one is the comparison of the prepared dosage form with the already established standard dosage form by similarity and dissimilarity factors. *In vivo—in vitro* (*IVIVC*) studies is the third type in which *in vivo* behavior can be predicted by using the *in vitro* data. BCS class II drugs categorized as a very potential candidate for *IVIVC* studies due to their *in vitro* profile as a rate-limiting step. Four levels i.e., A, B, C and multiple C of *IVIVC* can be developed after obtaining the *in vitro* and *in vivo* profile. Level A is very significant due to its point to point relationships of *in vitro* with *in vivo* data profile[8].

Level A showed a linear correlation and presented a point to point correlation between *in vitro* drug release studies and *in vivo* input rate. According to FDA guidelines, for the establishment of Level A correlation, formulations should be developed with altered drug release rates i.e. immediate (IR), intermediate (IntR) and slow release (SR) products or if drug release rate is condition independent then the single drug release rate is essential [9]. Level A correlation is of two-step method i.e. deconvolution step is followed by the assessment of the fraction of compound absorbed and dissolved. The detected fraction of drug absorbed is assessed by the numerical deconvolution procedure. The predicted fraction of drug absorbed is assessed using a detected fraction of drug dissolved. Now, these predicted drug absorbed values are used to estimate the predicted plasma concentrations by convolution method. The validity of the applied model is then assessed by computing the percentage prediction error (% PE) by comparing the difference between the predicted and observed values of several pharmacokinetic parameters i.e. C_max_& AUC_0-∞_. Sirisuth and Edington in 2002 estimated *IVIVC* model for naproxen and metoprolol [10]. Macha et al in 2009 reported a level A correlation for nevirapine formulations using WinNonlin *IVIVC* Toolkit and found < 10% average %PE for different pharmacokinetic parameters [11].

Nimesulide is a selective COX-2 inhibitor and recommended for inflammation, pain, and fever [12]. Nimesulide is available in 100mg tablets, microcapsules and in controlled release formulations [13]. It belongs to BCS class II and showed less solubility and high permeability.

The aim of the study was to establish a Level A *IVIVC* to illustrate the relationship between *in vitro* release and *in vivo* behavior of different Nimesulide formulations immediate release (IR) intermediate release (InR) and slow release (SR) formulations. Phoenix WinNonlin 7 and its *IVIVC* toolkit version 7.0 was used for the establishment of correlation. Furthermore, the validity of the applied model was tested by computing the prediction errors.

## 2. Materials & methods

### 2.1 Materials

Nimesulide was gifted from PharmEvo Pakistan. Microcrystalline cellulose (Avicel PH102) and carboxymethylcellulose (Ac-di-sol®) were purchased from FMC, Brussels, Belgium. Octadecanoic acid, magnesium stearate, and sodium lauryl sulphate were purchased from RDH Germany. Hydroxypropyl methylcellulose (Methocel K4M) was gifted from Colorcon, USA. Potassium dihydrogen phosphate, Hydrochloric acid, Sodium hydroxide, Sodium Chloride, sodium taurocholate, citric acid, lecithin, disodium hydrogen Phosphate, Triton X 100, sodium dihydrogen phosphate, glacial acetic acid, sodium hydroxide pellets, citric acid, ammonium acetate and HPLC grade, all were purchased from Merck, Darmstadt, Germany.

### 2.2. Fast, medium and slow release tablets

Tablets of three different release fashions were planned by using the central composite rotatable design (CCRD). Nine formulations F-IR1, F-IR2 and F-IR3 from immediate release F-InR1, F-InR2 and F-InR3 from intermediate release and F-SR1, F-SR2, F-SR3 were from the slow release were optimized (already published) [14–16] due to their excellent preformulation results. All the formulations reported in Table 1 were in weights ranges from 400 ± 20 mg were compressed by direct compression with single punch machine under the controlled conditions of humidity and temperature.

**Table 1:** Composition of 100mg Nimesulide tablets used for the development and validation of *in vitro in vivo* correlations (*IVIVC*).

Formulation	Code	Cro. Car. Na	Avicel	Mag. Ste	HPMC K4M	Tablet (mg)
Immediate Release	F-IR1	1.00	60.00	2.00	1.25	357.00
	F IR2	3.00	72.50	3.00	1.00	418.00
	F-IR3	1.00	83.00	1.00	1.00	444.00
Intermediate Release	F-InR1	0.00	52.23	2.61	5.12	339.84
	F-InR2	0.00	54.11	3.25	14.56	387.68
	F-InR3	0.00	67.42	4.82	12.53	439.08
Slow Release	F-SR1	0.00	34.16	3.61	31.06	375.32
	F-SR2	0.00	40.25	4.12	28.09	394.84
	F-SR3	0.00	48.25	3.61	32.15	435.4

### 2.3 Dissolution studies

Dissolution studies of marketed reference and compressed formulations F-IR1, F-IR2, F-IR3 from immediate release and F-InR1, F-InR2, F-InR3 from intermediate release F-SR1, F-SR2, F-SR3 from the slow release were performed using USP I and II apparatuses having different composition of dissolution medium. The different dissolution medium used were 0.1N HCl having pH 1.2, phosphate buffer solution of pH 4.5, 6.8, 7.4, fasted state simulated gastric fluid (FaSSGF), fasted state simulated intestinal fluid (FaSSIF) and fed state simulated intestinal fluid (FeSSIF)[17–19]. Six tablets of each formulation were placed in a 900 ml dissolution medium by setting the temperature limits 37 ± 0.5°C at 100 rpm. Effect of surfactant was analyzed by using 1–3% sodium lauryl sulphate (SLS) after dissolving it in phosphate buffer of pH 7.4. An aliquot of 5 ml medium was taken out from vessels at different time intervals and an equal volume was replaced by fresh medium. Syringe filter of 45 μm was used for filtration process and drug concentration was calculated by UV spectrophotometer at 297 nm each experiment was repeated three times.

### 2.4 *In vivo* studies

*In-vivo* absorption studies were a single centered, crossover, randomized, in four phases with two week washout period in male healthy volunteers (age: 18–27 years) under the complete guidelines of FDA (www.fda.gov). Weight range of the volunteers was between ±10 percent of the ideal body weight. All physical examinations and medical examinations were within normal limits, Allergy history was also analyzed which was found negative. Those volunteers whose, weights and heights were not in normal range, their diagnostic tests failed in case of medical examination in a clinical situation, smokers like having more than 10 cigarettes daily and any other addiction like alcohol or volunteers on special diet user i.e., spicy, vegetarian, rich diet were excluded from the study. The study was conducted under the supervision of principal investigator and physician in a private hospital in Karachi after getting the ethical approval from Pharmacy, Ethics Committee, Bahauddin Zakariya University Multan. Volunteers were initially informed about the pros and cons of the study and a written consent was taken in this regard. For the bioequivalence study, optimized formulations from immediate release F-IR1 were compared with standard marketed brand “Nimaran^®^” 100mg (Bosch Pharmaceutical). Optimized intermediate F-InR2 and slow release F-SR3 formulations were used for the *IVIVC* considerations with deconvolution approaches. These formulations were given to subjects in a fasting condition with 250 ml of water at 8:00 am in the morning, who already having an overnight fast condition of around 10 hrs. Time of dose administered treated as t = 0 hrs. No food was administered 4 hrs postdose. Regular breakfast was given after 4 hrs of the administered dose. Five mL of blood sample was drawn at various time points i.e. 0.5, 1, 2, 4, 8, 12 and 24 hr after the administration of F-IR1 and marketed brand, Nimaran. For intermediate release formulation, blood sampling time was found to be i.e. 0.5, 1, 2, 4, 8, 12, 24, 36 and 48 hr. For slow release formulation, sampling time was found to be 1, 2, 4, 8, 12, 24, 36 and 72 hr. Plasma was separated using centrifuge machine at 4000 rpm for 10 minutes. Samples were frozen at—20°C. Frozen samples were thawed and plasma drug concentrations were estimated after analyzing it using already validated HPLC the method (work already reported) [20].

### 2.5 *In vitro* data analysis

Two types of dissolution testers i.e., USP dissolution apparatus I (basket method) and USP dissolution apparatus II (Paddle method) (DT 600, Erweka, Heusenstamm, Germany) were used for *in-vitro* drug release studies. Twelve tablets of each formulation Reference brand (Nimaran), F-IR1, F-InR2, F-SR3 were placed in 900 ml dissolution medium by setting the temperature limits 37 ± 0.5°C at two different speeds 50 and 100 rpm in both dissolution apparatuses. Five mL aliquot was drawn from dissolution mediums having Nimaran tablets and F-IR1 formulation at 0.5, 1, 2, 4, 8, 12 hr. Similarly, the same volume of sample was taken for F-InR2 with the additional sampling time points of 24, 36 and 48 hrs. The concentration of Nimesulide from F-SR3 was observed after taking 5 mL of dissolution medium at 1, 2, 4, 8, 12, 24, 36 and 72 hr. The similarity between marketed and compressed immediate release formulation F-IR1was calculated by similarity factor *f*_2,_ applied by the following equation.

$$f_{2}=50\times \log\left\{{\left[1+(\frac{1}{n})\sum_{j-1}^{n}{\left|R_{j}-T_{j}\right|}^{2}\right]}^{-0.5}\times 100\right\}$$ (1)

Where n is the Number of samples, Rj and Tj are the Percentage release of reference and tests brands at different times respectively. Two formulations should be considered as similar if the *f*_2_ value is more than 50.

### 2.6 *In vivo* data analysis

Pharmacokinetic parameters of reference (marketed research brand) and compressed F-IR1, F-InR2 and F-SR3 were calculated using Kinetica^TM^ 4.4.1 (Thermoelectron, USA). Data was successfully fitted into oral two-compartment model and various other compartmental and non-compartmental parameters were calculated. Similarly, bioequivalence of F-IR1 and innovator products were carried out. Different bioequivalence attributes i.e., AUC_*last*_, AUC_*0-∞*_, AUC_*tot*_, C_*maxcalc*_ and T_*maxcalc*_ of both reference and test products were assessed using two-way ANOVA methods. Schirmann’s two-one sided *t-*test was applied for the verification of the bioequivalence assessment. Products bioequivalence were established by using 90% confidence interval (CI) values for reference and test products and the ratio of the values was targeted in the range of 0.8–1.25 for log-transformed and 0.8–1.20 for non-log transformed data respectively. For non-parametric assessment and carry-over effect, statistical software SPSS 20.0 (SPSS Inc.) was used.

### 2.7 Establishing *in vitro*- *in vivo* correlation (*IVIVC*)

Phoenix WinNonlin 7 and it's *IVIVC* toolkit version 7.0 (Certara USA, Inc., 100 Overlook Center, Suite 101, Princeton, NJ, USA) was used to estimate the absorption of Nimesulide. *In-vitro* release evaluation of Nimesulide was conducted using the Weibull model and curve shape was evaluated by shape factor β. Implicit, numeric and analytical are three different deconvolution method approaches used in previous literature among them numeric method with point area was used for the *IVIVC* studies. For the establishment *IVIVC* of level A the percentage prediction error of C_*max*_ and AUC was calculated.

$$C(t)=\sum_{n=0}^{{n=n}_{\tau max}}f(\tau_{n})C_{\delta }\;(t-\tau_{n})d\tau$$ (2)

Where, f(t): Input rate; C(t): Response; δ: Unit impulse; C_δ_: Unit impulse response; C_δ_(t): Impulse response (Khan et al., 2015).

### 2.8 Predictability error for *IVIVC* studies (Level A)

Internal (%PE) and external (%PE) prediction errors were used in the determination of IVIVC. Internal prediction for individual formulation was estimated by its C_*max*_ and AUC values. Prediction error was used to assess the comparison between observed and predicted bioavailability. In the present study, external prediction error was not used because the values of internal % PE of AUC and C_*max*_ were within the adequate limits. For the *IVIVC* predictability, the accepted limits for %PE for C_max_ and AUC were ≤10 [9]. The %PE_AUC_ and % PE_Cmax_ can be calculated using the following equations:

$$\%{PE}_{AUC}=\frac{\left({AUC}_{observed}–{AUC}_{Predicted}\right)}{{AUC}_{Observed}}\times 100$$ (3)

$$\%{PE}_{Cmax}=\frac{({Cmax}_{observed}–{Cmax}_{Predicted})}{{Cmax}_{Observed}}\times 100$$ (4)

## 3. Results and discussion

### 3.1 *In vitro* dissolution studies

**Fig 1:**
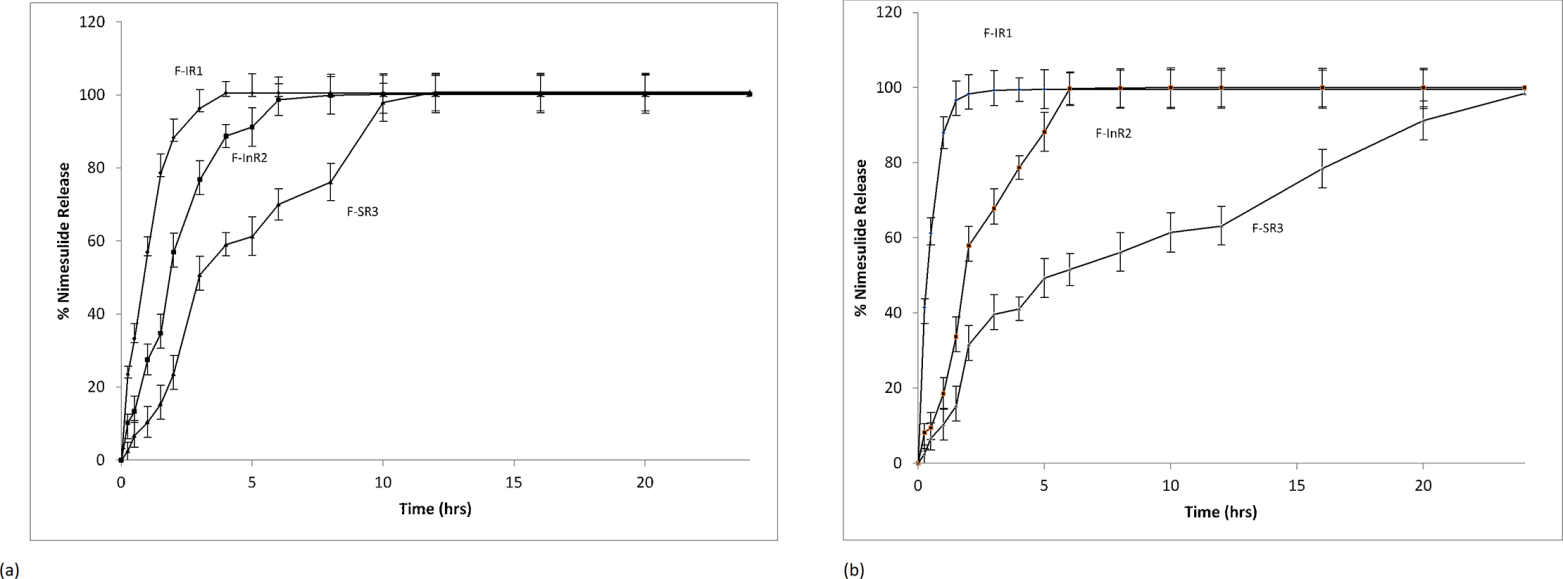
Time versus % *in vitro* Nimesulide release from Immediate (F-IR1), Intermediate (F-InR2) and slow release (F-SR3) Nimesulide 100 mg tablets in a) Fasted state of gastric and (b) in the Intestinal fluid.

More than 85% of Nimesulide was released from F-IR1, F-IR2 and F-IR3 within 60 minutes. pH-dependent percentage of Nimesulide released was observed in the different dissolution medium. Comparatively less amount of drug release was observed at pH 1.2 and 4.5 medium, which was may be due to weak acidic nature and pKa values of HPMC K4M. It was increased pH 6.8 and 7.4, may be due to its dissociation and micelle formation after pH 6.5 of Nimesulide. A higher percentage of drug was released with 1% SLS already reported [8]. In F-InR1, F-InR2 and F-InR3 the average drug release were nearly 10% reduced in comparison to immediate release formulations. Reason may be due to concentration of HPMC which showed the controlled release behaviour when used in the range of 5–15% [21–23]. Hydrophilic polymer HPMC K4M showed inverse relationship with release rate of Nimesulide due to the presence of carboxylic group and swelling nature [21, 24]. In case of slow release formulations higher concentration of HPMC K4M (15–35%) along with poor solubility of Nimesulide further decreased 10% release rate as compared to the intermediate release formulation. Fig 1 showed the percentage release of Nimesulide 100 mg in fasted gastric and intestinal fluids. Immediate release formulations were compared with innovator brand and results revealed that F-IR1 showed highest similarity (*f*_2_) values i.e., 85.685, 90.177, 73.559 and 68.559% at pH 1.2, 4.5, 6.8 and 7.4 respectively. Similarity factor of F-InR1 and F-InR2 after comparing with F-InR3 which was selected as a reference formulation due to its excellent physicochemical properties were 55.462 and 59.672 in pH 1.2, 62.695 and 67.337 in pH 4.5, 57.521 and 74.900 in pH 6.8 and 60.188 and 80.791 in pH 7.4. Similarly, difference factor of F-InR1 and F-InR2 after comparing with F-InR3 were 12.947 and 10.005 in pH 1.2, 6.331 and 5.167 in pH 4.5, 8.999 and 7.445 in pH 6.8, 13.042 and 7.531in pH 7.4. Similarity (*f*_2_) values of F-SR1 and F-SR3 with F-SR2 were 76.891 and 68.195 in pH 1.2, 72.748 and 55.309 in phosphate buffer pH 4.5, 74.900 and 58.828 in slightly acidic pH 6.8, 80.791 and 45.697 in slightly basic pH 7.4. Formulations F-IR1 from immediate release, F-InR2 from intermediate release and F-SR3 from the slow release were selected for further pharmacokinetic studies due to their excellent physicochemical and quality control attributes. Table 2 showed the correlation (r^2^) values of different release formulations in different dissolutions apparatuses and dissolution mediums.

**Table 2:** Correlation (r^2^) values using Weibull model for Nimesulide 100 mg tablets at different conditions of dissolution apparatus and different composition of dissolution mediums.

Formulation	pH 1.2	pH 4.5	pH 6.8	pH 7.4	FaSSGF	FeSSIF	FaSSIF
USP Dissolution apparatus I with 100rpm							
Reference Product	0.897	0.878	0.888	0.894	0.914	0.884	0.842
F-IR1	0.889	0.889	0.887	0.902	0.812	0.784	0.812
F-InR2	0.885	0.912	0.895	0.912	0.842	0.841	0.874
F-SR3	0.998	0.912	0.999	0.999	0.884	0.910	0.841
USP Dissolution apparatus II with 100rpm							
Reference Product	0.909	0.934	0.933	0.995	0.874	0.898	0.826
F-IR1	0.996	0.988	0.995	0.988	0.888	0.876	0.888
F-InR2	0.900	0.922	0.911	0.914	0.733	0.940	0.915
F-SR3	0.989	0.990	0.997	0.993	0.884	0.873	0.986
USP Dissolution apparatus II with 50rpm							
Reference Product	0.785	0.898	0.888	0.952	0.841	0.884	0.914
F-IR1	0.841	0.875	0.899	0.914	0.774	0.821	0.845
F-InR2	0.712	0.897	0.912	0.8965	0.812	0.814	0.842
F-SR3	0.999	0.998	0.956	0.9458	0.741	0.941	0.924

### 3.2 *In vivo* pharmacokinetic studies

For the log-transformed values, the geometric mean ratio of C_max_ calculated for reference and test was 0.995 μg/ml while geometric mean C_max_ values were 6.087 ± 1.072 and 6.060 ± 1.073 μg/ml respectively. ANOVA results were found to be in significant range. P values of formulations and periods were insignificant whereas sequence and subject were found to be significant. The 90% confidence interval (CI) for C_*max*_ (observed) were (99.17–100.3%) and for C_*max*_ (calculated) were (99.3–99.7%). Similarly, values of geometric mean for T_*max*_ (observed) for reference and test formulations were (1.3812 ± 1.1745 and 1.366 ± 1.1724 hr). In ANOVA the formulations, subject and sequence were found to be insignificant having geometric ratio of 0.989. The 90% confidence of interval values for T_*max*_ were 98.198 to 99.658%. Oberved values of AUC_*ext*_ were 100.95 to 104.91% and AUC_*total*_ were 99.88 to 100.08%. For AUC_*ext*_ and AUC_*total*_, values for geometric mean values for both reference and test products were (0.110 ± 1.378 and 0.1141 ± 1.363) and AUC_*total*_ were (23.6451 ± 1.02917 and 23.640 ± 1.028) respectively. For non -log transformed data, values of geometric mean for C_*max*_ (observed) of reference and test products were (6.01084 ± 1.19275 and 5.99586 ± 1.19615), while C_*max*_ (calculated) were (6.087 ± 1.072 and 6.060 ± 1.07314). Values of AUC_*total*,_ *T*_*max*_ and AUC_*last*_ were found within the required limits. For non-log transformed data, the 90% confidence intervals (CI) values for C_*max*_ (observed) (99.25–100.32%), C_*max*_ (calculated) (99.33–99.69%), T_*max*_ (98.188–99.834%) AUC_*total* (_99.88–100.08%). In this study values of C_*max*_, AUC and T_*max*_ of F-IR1and reference product were statistically lies in the acceptable limits in both (log and non-log transformed) data. Bernareggi et al., in 1998 found the similar results after conducting bioequivalence studies on granules, tablets, and suspension [13].

### 3.3 Development of *IVIVC* correlations

**Fig 2:**
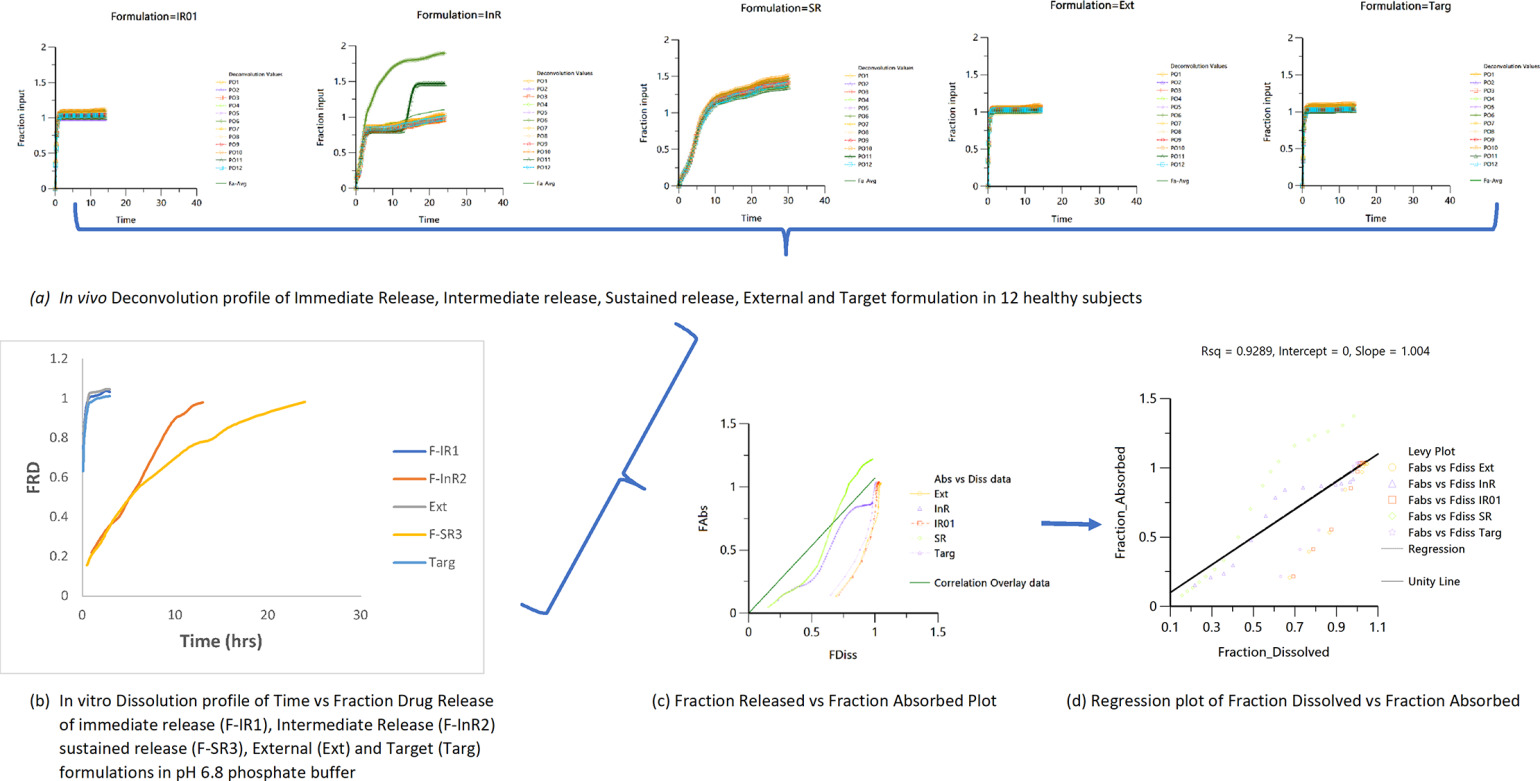
Diagrammatic flow of level A In-Vito In-Vivo Correlations (*IVIVC*) using the deconvolution techniques.

Multiple formulations of F-IR1, F-InR2 and F-SR3 were designed and developed using HPMC as a rate controlling polymer. Data indicated that the as the concentration of HPMC reduces the drug release rate. In this study, *in vitro* release assessment were conducted at several dissolution media i.e. pH 1.2, 4.5, 6.8, 7.4; FaSSGF, FaSSIF and FeSSIF using USP dissolution Apparatus I (at 100 rpm) and Apparatus II (at 50 and 100 rpm). Fig 2 showed the development of Level A *IVIVC* using the deconvolution method for Nimesulide 100 mg tablets. At pH 1.2 (50 rpm of USP II and 100 rpm of USP II and I), numeric values of % PE of AUC_*inf*_ were found to be (6.788, 9.587 and 8.974) and C_*max*_ (6.055, -2.414 and 1.531) respectively. Similarly, numeric values of %PE of AUC_*inf*_ at pH 4.5 and pH 6.8 were found to be (9.156, 9.207 and 9.355) and (2.584, 2.978 and 2.472) and C_*max*_ values were (-4.745, -3.276 and -5.526) and (2.584,0.573 and 4.692) respectively. At pH 7.4 (50 rpm of USP II and 100 rpm of USP II and I), consecutive numeric values of AUC_*inf*_ andC_*max*_were found to be (4.350, 7.173 and 7.094; 5.621, 3.055 and -2.471). Similarly, values of %PE of AUC_*inf*_ were consecutively found to be (9.372, 9.542 and 9.150); (9.900, 9.167 and 8.147) and (9.886, 8.212 and 9.130)and C_*max*_ (-7.228, 8.957 and -6.680); (3.695, 7.573 and 9.161) and (-29.059, -6.085 and -7.061) at FaSSGF, FaSSIF and FeSSIF (50 rpm of USP II and 100 rpm of USP II and I) as shown in Table 3. Eddington et al., in 1998 reported values of internal prediction error (%PE) of C_*max*_ and AUC_*last*_ of metoprolol tartrate (ER) tablets [25].

**Table 3:** Deconvolution approaches and internal prediction error (%) in different dissolution mediums.

		**pH 1.2**	**pH 4.5**	**pH 6.8**	**pH 7.4**	**FaSSGF**	**FeSSIF**	**FaSSIF**
**Ref**	AUC_*inf*_	-4.598	-2.651	5.204	10.991	7.844	3.214	15.243
	C_max_	11.130	10.610	9.541	7.965	7.433	14.234	12.632
**F-IR1**	AUC_*inf*_	7.586	-0.869	1.324	17.284	9.561	15.178	14.336
	C_max_	2.982	5.677	2.647	-7.315	4.342	8.806	3.5833
**F-InR2**	AUC_*inf*_	-27.182	-27.96	-33.203	-11.88	19.201	-35.503	-24.73
	C_max_	14.568	14.823	13.245	6.125	12.796	15.962	13.460
**F-SR3**	AUC_*inf*_	1.134	9.939	1.421	1.601	8.358	14.287	13.82
	C_max_	14.523	13.682	13.854	12.425	13.254	15.126	12.874
**Average**	AUC_*inf*_	11.967	12.924	11.983	10.255	12.373	15.124	13.631
	C_max_	-4.598	-2.651	5.204	10.991	7.844	3.214	15.243

For AUC_*inf*_ and C_*max*_, the %PE value of each formulation was found to be < 15% while the average values were < 10%. The low values of prediction errors demonstrate that the correlation model is projecting the *in vivo* response of each formulation. Thus indicated a valid Level A *IVIVC* according to the FDA guidance document [26]. External (%PE) was not required because individual values of internal (%PE) error of C_*max*_ and AUC_*last*_ were not >15. As compared with other nonlinear models, the Weibull model was considered to be the best fit for *in vitro*- *in vivo* data. Estimation of regression values (r^2^) is significantly important for point to point correlation. Correlation (r^2^) values after comparison of *in-vivo* absorbed *v*s. *in-vitro* drug dissolved values at different dissolution media were successfully calculated. Linear correlation found at pH 6.8 and 7.4 i.e. r^2^ = 0.999. Jantratid *et a*l in 2006 reported excellent value of correlation i.e. r^2^ = 0.968 for cimetidine tablets to develop *IVIVC* model [27]. The reported results are quite similar to the Tandt *et al*., in 1995 reported the *IVIVC* correlation at phosphate buffer pH 6 [28]. Results of the present study indicated that *IVIVC* correlation was obtained at pH 6.8 and pH 7.4 showed good correlation Also, internal prediction error (%PE) of AUC_*inf*_ and C_*max*_ were calculated as presented in Table 3. In the previous literature, the fed state of gastric and intestinal medium also considered as the best for correlation[18, 19]. Leu et al in 2008 established the *IVIVC* model for hemibenzathine and demonstrated that best medium was found to be fed state to predict AUC and C_*max*_ showed < 10% prediction error [29].

## Conclusion

In this study, a Level A *IVIVC* was established which demonstrate the relationship between *in vivo* absorption data and *in vitro* release data for all Nimesulide products. Results of internal validation were found to be within the adequate limits indicating the prediction of correlation models.

## Supporting information

S1 File “Compartive Excel Data of Test and Reference Brand.xlsx”. (XLSX)

S2 File “In vivo data IVIVC.xlsx”. (XLSX)
